# Comparison of TFL and high-power Ho: YAG lasers with multiple pulse modulations: an in vitro study

**DOI:** 10.1007/s00345-026-06514-x

**Published:** 2026-06-18

**Authors:** Alejandra Bravo-Balado, Antoni Sánchez-Puy, Paula Izquierdo, Pietro Diana, Alberto Piana, Damiano Stracci, Paola Arena, Sílvia Gràcia Garcia, Andrés Koey Kanashiro, Félix Millán, Francisco Sánchez-Martín, Cristina Esquinas, Oriol Angerri, Olivier Traxer, Joan Palou, Esteban Emiliani

**Affiliations:** 1 Department of Surgery and Morphological Sciences, Universitat Autónoma de Barcelona, Barcelona, Spain; 2 Department of Urology, Fundació Puigvert, Barcelona, Spain; 3 Endolase Lab, GRC20-Sorbonne University and PIMM-Arts et Métiers Paris Tech, Paris, France; 4 Service d’Urologie, Assistance-Publique Hôpitaux de Paris, Hôpital Tenon, Sorbonne Université, 4 rue de la Chine, Paris, 75020 France; 5 Urinary Lithiasis Laboratory Unit, Fundació Puigvert, Barcelona, Spain; 6 Faculty of Nursing, Department of Public Health, Mental Health and Maternal and Child Health Nursing, University of Barcelona (UB), Barcelona, Spain; 7 Department of Urology, NYU Langone Health, New York, NY USA

**Keywords:** Ablation Volume, Ho:YAG, In vitro, Pulse modulation, TFL

## Abstract

**Purpose:**

To compare the stone ablation volume (AV) achieved by thulium fiber laser (TFL) and high-power Holmium: YAG (Ho: YAG) lasers with MOSES™, Virtual Basket™ (VB™), Vapor Tunnel™ (VT™), and Magneto pulse-modulation technologies in an artificial stone model.

**Methods:**

BegoStone phantoms (15:6 “powder-to-water” ratio) were used. A motorized arm applied the laser at a constant speed (0.7 mm/s). Laser settings included 1.5 J × 5 Hz, 1 J × 20 Hz, and 0.3 J × 50 Hz, with five 21-mm cuts per setting. AV was calculated from width and depth measurements using an optical microscope. ANOVA was performed.

**Results:**

Among 750 measurements, TFL-200 μm achieved the highest AV in most settings. At 1.5 J x 5 Hz, TFL-200 μm (15.9 mm³) significantly outperformed MOSES™ (LP: 11 mm³, SP: 10.4 mm³, MD: 9.7 mm³) and Magneto (9.1 mm³) (*p* < 0.05). At 1 J × 20 Hz, TFL-200 μm (21.2 mm³) exceeded all technologies (*p* < 0.001). At 0.3 J x 50 Hz, TFL-200 μm (8.9 mm³) outperformed MOSES™ MD (3.8 mm³) and Magneto (4.9 mm³), *p* < 0.05. TFL-150 μm showed comparable AV to Ho: YAG lasers across settings, except at 1 J x 20 Hz, where it was lower than TFL-200 μm and CyberHo150 SP and VT™ (*p* < 0.05), primarily due to lower depth of fissure (DOF) while width of fissure (WOF) remained comparable. At 0.3 J × 50 Hz, TFL-150 μm (6.7 mm³) demonstrated lower AV than CyberHo150 SP (10.9 mm³; *p* = 0.003), but higher AV than MOSES™ MD (3.8 mm³; *p* = 0.034).

**Conclusions:**

TFL-200 μm achieved higher AV than Ho: YAG in most settings, but statistical significance was observed in one-third of comparisons. TFL-150 μm demonstrated similar AV to Ho: YAG, except in cases where Ho: YAG had higher DOF. TFL-150 μm has known advantages in terms of irrigation, visibility, ureteroscope tip deflection, and potential for miniaturization of flexible ureteroscopy devices and aspiration methods, supporting its potential applicability for dusting strategies while maintaining comparable AV.

## Introduction

The race for the most efficient laser technology in endourology has never been more competitive. For years, the Holmium: YAG (Ho: YAG) laser has been the gold standard for lithotripsy, delivering reliable stone fragmentation and dusting [1, 2]. However, the landscape of laser lithotripsy is evolving rapidly. The thulium fiber laser (TFL) emerged as a strong contender, offering finer dusting, reduced retropulsion, and improved efficiency [3, 4]. Meanwhile, advancements in Ho: YAG technology, such as the Magneto Technology (Magneto), incorporate pulse modulation to optimize performance by reducing peak power due to an extended pulse duration (up to 2000 µs), potentially leading to improved dusting properties. Other Ho: YAG innovations, such as the MOSES™ technology (MOSES™), Virtual Basket™ (VB™), and Vapor Tunnel™ (VT™), have been more extensively studied, with evidence supporting their benefits over conventional pulse modes [2, 5–9]. MOSES™ consists of two sub-pulses with different peak powers. Similarly, VB™ uses a double-pulse system in which the first pulse creates a vapor bubble and the second one propagates through it, whereas VT™ acts as a single extended pulse. These technologies were developed to reduce retropulsion.

Despite these advancements, results remain controversial. Moreover, the influence of laser fiber diameter on lithotripsy efficiency is still a matter of debate, with contradicting results [10, 11].

In this in vitro study, we compared the ablation volume (AV) achieved by TFL and next-generation pulse-modulated Ho: YAG technologies, including MOSES™, VB™, VT™, and Magneto, using an artificial stone model. We also evaluated the influence of TFL fiber diameter (150 μm vs. 200 μm) and compared these findings with 200 μm Ho: YAG laser fibers.

## Materials and methods

### Experimental setting and stone phantoms

**Image 1A:**
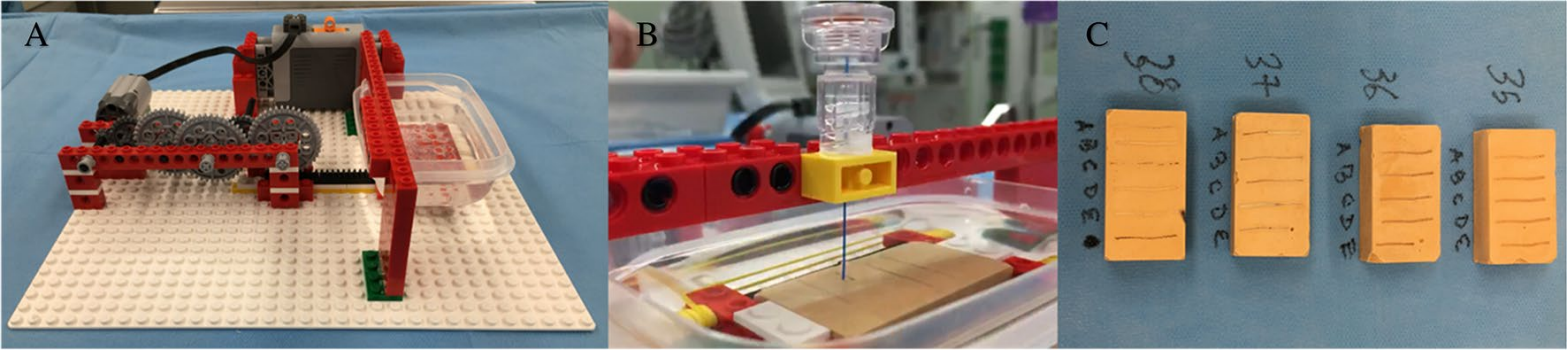
**A** An automated arm performs laser cuts on the BegoStone phantoms. A container filled with 0.9% saline solution (right side of the picture) rests. **B** With the help of a motor, the automated arm moves in a straight line at a constant speed (0.7 mm/s). **C** Five 21 mm linear cuts for each setting were made, measuring width and depth in order to calculate ablation volume.

We used BegoStone phantoms (25 × 50 mm, 15:6 “powder-to-water” ratio) to simulate urinary soft stones. The 15:6 ratio was selected to increase experimental sensitivity, as previous studies have shown no significant differences in ablation patterns between hard and soft BegoStones when using pulse-modulated laser settings [6, 12]. After production, phantoms were air-dried for 48 h at room temperature to minimize heterogeneity. The phantoms were then submerged in 0.9% saline solution (Image 1A). A custom-built automated system using Lego Technic™ was developed, consisting of a motorized platform moving the phantom linearly at 0.7 mm/s (Image 1B). We performed all experiments using a fiber-to-stone distance of 0.5 mm, measured with a Vernier caliper, to ensure reproducibility. The laser fiber was stabilized using a Tuohy-Borst valve. In a few cases, the fiber tip became trapped in the crater of a sliding stone; in those cases, the experiment was excluded and repeated. A similar mechanism was used by Kronenberg and Traxer in 2014 [1].

### Laser sources

We evaluated four laser sources: 60 W TFL (Fiber Dust®, Quanta System©, Italy), 150 W Ho: YAG (Cyber Ho and Cyber Ho Magneto, Quanta System©, Italy) and 120 H Ho: YAG with MOSES™ technology (Lumenis Pulse™ 120 H, Yokne’am Illit, Israel).

Three energy-frequency combinations were tested: 1.5 J × 5 Hz, 1 J × 20 Hz and 0.3 J × 50 Hz. For Ho: YAG lasers, the following pulse modulations were applied: short pulse (SP), long pulse (LP), MOSES™ Contact (MC), MOSES™ Distance (MD), VT™, VB™, and Magneto. For TFL, only SP was used, as evidence suggests TFL with SP achieves superior ablation compared to TFL with LP, which has an extremely low peak power not suited for laser lithotripsy [13, 14]. Certain pulse modulations were not available for specific settings (e.g., VT™ at 1.5 J × 5 Hz and 0.3 J × 50 Hz); therefore, those experiments were not conducted. The choice of laser parameters was based on a combination of clinically relevant scenarios, device platform characteristics, and prior literature, encompassing both fragmentation and dusting techniques [5, 15–17].

### Physical characteristics of lasers

1. Laser pulse characteristics
  Ho: YAG is a 2,120 nm wavelength solid-state pulsed laser, highly absorbed by water, producing primarily photothermal effects, although cavitation and photomechanical mechanisms may also contribute to stone ablation and fragmentation [18, 19]. It emits energy in single pulses with pulse durations typically ranging from 200 to 1,200 µs, depending on pulse modulation, and extending up to 2,000 µs with newer pulse-modulated technologies such as Magneto. SP and LP were among the earliest pulse modulation modes developed for Ho: YAG lasers. SP delivers a rapid, high-peak-power pulse of approximately 150–350 µs [20], generating greater retropulsion and making it more suitable for stone fragmentation. Conversely, LP delivers a longer pulse of up to 1,200 µs, thereby reducing retropulsion and making it better suited for dusting.

In 2017, Lumenis® (Yokne’am Illit, Israel) introduced MOSES™, which splits the pulse into two sub-pulses with different peak powers. The first sub-pulse creates a vapor bubble, allowing the second sub-pulse to travel through it with minimal energy loss, improving energy delivery to the stone. Two MOSES™ modes were developed:

- MC – optimized for 1 mm working distance.
- MD – optimized for 2 mm working distance [21–23].

Additional pulse modulation technologies were later introduced:

- VB™ – a double-pulse system, where a first pulse creates the vapor bubble, and a second pulse propagates through it, potentially reducing stone movement.
- VT™ – a single, extended pulse designed to limit retropulsion by maintaining lower peak power.

Precise pulse duration values have not been consistently reported for these pulse modulations.

- Magneto – an ultra-long pulse; it has been reported to operate with prolonged pulse durations (up to ~ 2,000 µs). However, precise pulse duration values for specific modes are not consistently detailed in the peer-reviewed literature.

In contrast to Ho: YAG, TFL uses a silica fiber doped with thulium ions and excited by diode pumps, allowing energy generation at a wavelength of 1940 nm [24], nearly matching water’s absorption peak, resulting in water absorption approximately four times greater than that of Ho: YAG [18]. TFL pulse characteristics differ substantially from those of Ho: YAG systems. It operates using a quasi-continuous emission composed of high-frequency micro-pulses aggregated into a “macro-pulse”. These pulse durations are longer, although not directly comparable to Ho: YAG pulses, ranging from hundreds of microseconds to several milliseconds (200–12,000 µs) [25]. This emission profile results in a more homogeneous energy delivery over time [24].

2. Peak power characteristics
  Peak power in Ho: YAG systems varies according to pulse duration and modulation, and can reach several kilowatts in SP mode. Peak power progressively decreases in LP, VB™, and VT™ modes and is further reduced in Magneto due to pulse elongation. In contrast, TFL generates substantially lower peak power, often in the range of several hundred watts, resulting in a more homogeneous photothermal energy delivery. Higher peak power pulses increase cavitation bubble expansion and collapse, enhancing fragmentation but also increasing retropulsion. Higher peak power pulses increase cavitation bubble expansion and collapse, promoting fragmentation but also increasing retropulsion. Conversely, lower peak power with sustained energy delivery, as observed with TFL and extended-pulse Ho: YAG modalities, favors gradual material removal and finer particle generation, supporting improved dusting efficiency [24, 25].
3. Laser fiber diameter
  All experiments were performed using 200 μm fibers; TFL was additionally tested with a 150 μm fiber. Although some modern Ho: YAG platforms may theoretically accommodate smaller fiber diameters, to our knowledge, no commercially available Ho: YAG systems currently support fibers smaller than 200 μm. This limitation is thought to be related to the intrinsic physical characteristics of Ho: YAG lasers. Unlike TFL, Ho: YAG systems generate higher peak power and more pronounced cavitation effects, which may result in inefficient energy coupling in small-core fibers and an increased risk of fiber degradation [26].

### Ablation volume assessment

For each laser setting, five linear fissures (21 mm each) were created; this length was standardized across all experiments to ensure experimental consistency. Width (WOF, µm) and depth (DOF, µm) of fissures measurements were obtained at three points per fissure, totaling 15 measurements per setting. Measurements were performed using an Axiolab RE optical microscope, with depth assessed using a depth-of-focus technique, previously described and validated in the literature [27].

Estimated mean AV (mm³) was calculated as: mean WOF × fissure length × mean DOF.

### Statistical analysis

WOF, DOF, and AV were analyzed independently. The width-to-depth ratio was additionally calculated to evaluate whether ablation patterns were predominantly wider or deeper.

Data normality was assessed before conducting ANOVA to compare ablation volumes across laser settings. When significant differences were identified (*p* < 0.05), post-hoc comparisons were performed using Tukey’s HSD test. Statistical analyses were conducted using R version 4.2.

## Results

A total of 750 WOF and DOF measurements were obtained across all laser settings and pulse modulations.

Tables 1 and 2, and 3 summarize WOF, DOF, and AV results for the three tested settings: 1.5 J × 5 Hz, 1 J × 20 Hz, and 0.3 J × 50 Hz, respectively. Each table compares TFL-200 μm and TFL-150 μm fibers with the different Ho: YAG pulse-modulation modalities.

**Table 1:**
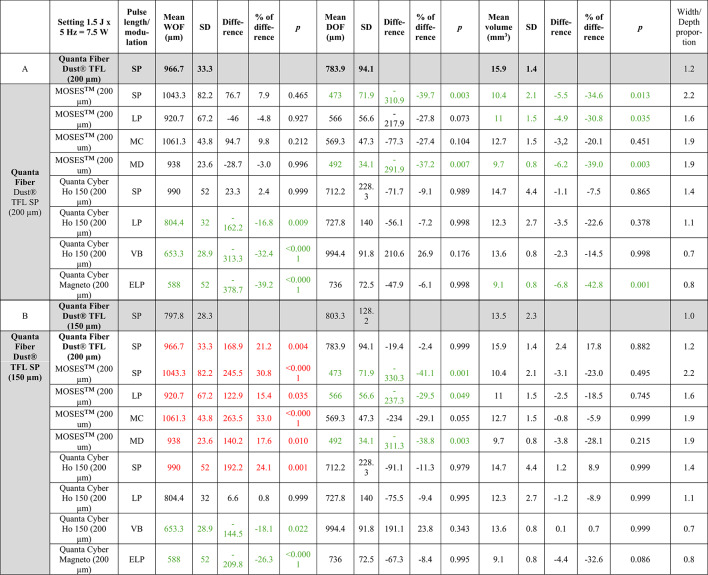
Comparisons between TFL and Ho: YAG lasers using 1.5 J x 5 Hz

*SD* standard deviation, *SP* short pulse, *LP* long pulse, *MC* Moses Contact, *MD* Moses Distance, *ELP* extra-long pulse, *VB* Virtual BasketTM, *WOF* width of fissure, *DOF* depth of fissure. Green-highlighted values indicate statistically significant superiority of TFL, whereas red-highlighted values indicate statistically significant superiority of the comparator Ho:YAG modality (p < 0.05). Non-colored values indicate no statistically significant difference.

**Table 2:**
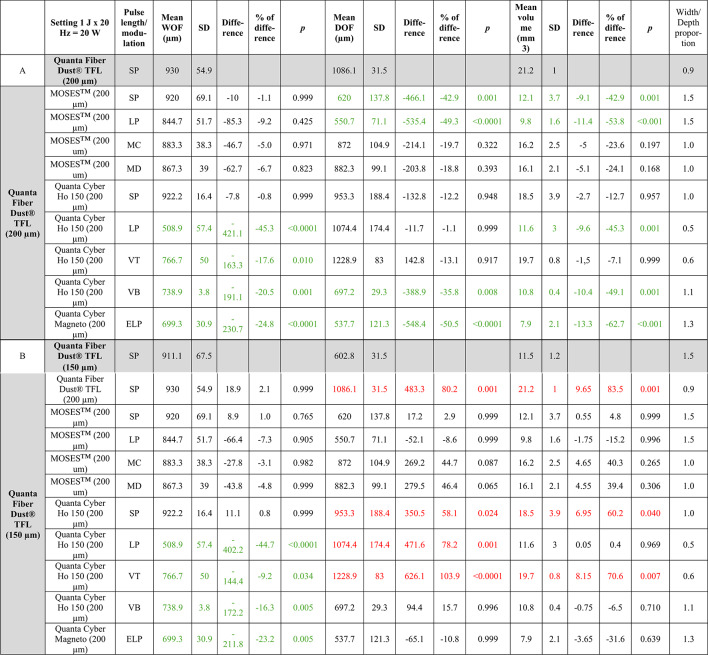
Comparisons between TFL and Ho: YAG lasers using 1.5 J x 5 Hz

*SD* standard deviation, *SP* short pulse, *LP* long pulse, *MC* Moses Contact, *MD* Moses Distance, *ELP* extra-long pulse, *VT* Vapour TunnelTM, *VB* Virtual BasketTM, *WOF* width of fissure, *DOF* depth of fissure. Green-highlighted values indicate statistically significant superiority of TFL, whereas red-highlighted values indicate statistically significant superiority of the comparator Ho:YAG modality (p < 0.05). Non-colored values indicate no statistically significant difference.

**Table 3:**
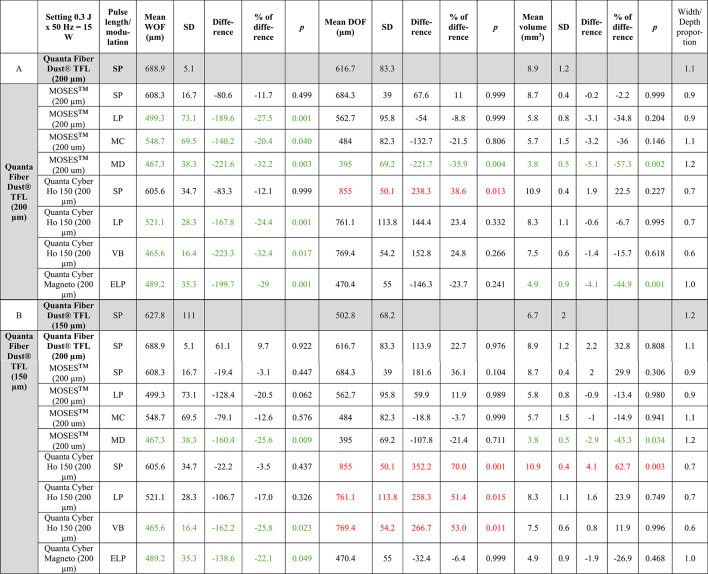
Comparisons between TFL and Ho: YAG lasers using 0.3 J x 50 Hz

*SD* standard deviation, *SP* short pulse, *LP* long pulse, *MC* Moses Contact, *MD* Moses Distance, *ELP* extra-long pulse, *VB* Virtual BasketTM, *WOF* width of fissure, *DOF* depth of fissure. Green-highlighted values indicate statistically significant superiority of TFL, whereas red-highlighted values indicate statistically significant superiority of the comparator Ho:YAG modality (p < 0.05). Non-colored values indicate no statistically significant difference.

### TFL with 200 μm fiber

When comparing TFL with a 200 μm fiber with Ho: YAG lasers, TFL consistently demonstrated higher mean AV across most settings.

At 1.5 J × 5 Hz, TFL (15.9 mm³) outperformed the MOSES™ LP: 11 mm³ (*p* = 0.0132), SP: 10.4 mm³ (*p* = 0.0354), MD: 9.7 mm³ (*p* = 0.0032), and Magneto: 9.1 mm³ (*p* = 0.0009). Interestingly, in the case of Magneto, this difference was attributable to the greater WOF achieved by TFL (966.7 μm vs. 588 μm), rather than differences in DOF (Table 1A).

A similar trend was observed at 1 J × 20 Hz, where TFL (21.2 mm³) significantly outperformed MOSES™ SP: 12.1 mm³ (*p* = 0.0004), LP: 9.8 mm³ (*p* < 0.0001); Quanta Cyber Ho 150: LP: 11.6 mm³ (*p* = 0.001), VB™: 10.8 mm³ (*p* = 0.0003) and Magneto: 7.9 mm³ (*p* < 0.0001). Again, AV differences were primarily driven by greater WOF rather than DOF, except for MOSES™ SP and LP modes, in which the AV difference was associated with greater DOF achieved by TFL (Table 2A).

At 0.3 J × 50 Hz, TFL consistently produced greater WOF, while DOF remained comparable across lasers. In some cases, Ho: YAG lasers exhibited greater DOF, particularly Quanta Cyber Ho 150 SP (855 μm vs. 616.7 μm). Consequently, fewer statistically significant differences in AV were observed: TFL (8.9 mm³) demonstrated higher AV than MOSES™ MD (3.8 mm³, *p* = 0.002) and Magneto (4.9 mm³, *p* = 0.0016) (Table 3A).

### TFL with 150 μm Fiber

When comparing TFL-150 μm with other laser systems, results varied according to laser settings.

At 1.5 J × 5 Hz, TFL-150 μm produced lower WOF (797.8 μm) compared with most lasers, except for Quanta Cyber Ho 150 VB™ and Magneto (653.3 μm and 588 μm, respectively). However, TFL-150 μm achieved greater DOF than MOSES™ SP, LP and MD. Consequently, no significant differences in ablation volume were found (Table 1B).

At 1 J × 20 Hz, TFL-150 μm generated greater WOF compared to Quanta Cyber Ho 150 SP, LP, VT™, VB™ and Magneto, whereas mean DOF remained similar or even lower than that of other lasers using 200 μm fibers. Consequently, greater AV was observed with TFL-200 μm and Quanta Cyber Ho 150 SP, LP and VT™, which were associated with greater DOF (Table 2B).

At 0.3 J × 50 Hz, few significant differences in AV were found. Notably, MOSES™ MD demonstrated lower mean AV, whereas Quanta Cyber Ho 150 SP achieved higher AV accompanied by greater DOF (Table 3B).

## Discussion

Currently, the choice of laser for stone lithotripsy depends on multiple factors, including availability, desired surgical technique (fragmentation vs. dusting), and stone characteristics [28]. Ho: YAG lasers have long been the gold standard due to their versatility in both fragmentation and dusting techniques. However, the emergence of TFL has introduced new possibilities, particularly in dusting, as it generates finer dust particles [4]. Several studies suggest that, at equal settings, TFL achieves a higher ablation rate and volume than Ho: YAG [3, 18, 25, 29].

In this study, we conducted a comparative analysis of stone AV across four available lasers approved for lithotripsy featuring novel pulse modulation capabilities. We also assessed the impact of fiber size, comparing 150 μm and 200 μm fibers for TFL and 200 μm fibers for Ho: YAG. The use of an automated experimental setup ensured a standardized ablation technique and experimental reproducibility.

When comparing 200 μm fibers, TFL achieved higher stone AV in most comparisons against Ho: YAG across different laser settings. However, the difference was statistically significant in only a third of cases. When using 150 μm fibers, the results were more comparable across different lasers and settings, with only a few exceptions. These differences were primarily due to lower DOF with TFL-150 μm rather than lower WOF.

Previous studies have evaluated the performance of pulse-modulation Ho: YAG lasers. Sánchez-Puy et al. [21] published a meta-analysis in 2022 reporting that MOSES™ was the most extensively studied modality, showing improved lithotripsy performance, shorter operative times, and reduced retropulsion compared to conventional pulses. However, other authors argue that ablation rates are comparable between MOSES and LP Ho: YAG, with both being inferior to TFL [30]. Similarly, Bozzini et al. [31] conducted a randomized controlled trial on the Cyber Ho 150 laser with and without VB™. Their findings showed that VB™ technology was associated with shorter fragmentation times and reduced stone retropulsion during laser lithotripsy. Basulto-Martínez and colleagues also compared the ablation rates of Ho: YAG and TFL with different setting combinations, finding that TFL outperformed Ho: YAG in most settings, although the ablation rate of Ho: YAG with VB™ became comparable to that of TFL [18]. Regarding Magneto, to date, there are no in vitro studies evaluating its lithotripsy performance. However, a retrospective comparative in vivo study including 127 patients reported similar ablation efficacy, efficiency, laser energy consumption, and stone-free rates between TFL and Magneto [32]. The authors emphasized that larger randomized studies are required to confirm these findings.

The impact of fiber size on AV remains controversial. Older studies suggested that larger fibers fragment larger volumes, which may hold true for Ho: YAG lasers [33]. However, more recent studies using TFL indicate different results [29]. Our study found that fiber diameter had little influence on AV for TFL in most comparisons. An exception was observed at 1 J × 20 Hz, where Quanta Fiber Dust® TFL SP and Quanta Cyber Ho 150 SP and VT™ exhibited higher AV, as well as at 0.3 J × 50 Hz with Quanta Cyber Ho 150 SP. As suggested by previous authors, these differences may relate to variations in energy density at the stone surface, particularly under low-power settings where the ablation threshold may not be consistently reached [33, 34].

Although fiber size had only a modest impact on AV in our study, smaller fibers (150 μm) may offer advantages for endoscopic lithotripsy, including improved irrigation flow, visibility, ureteroscope deflection, and compatibility with miniaturized f-URS and aspiration systems [10, 11]. Previous studies have also suggested that smaller TFL fibers may facilitate improved dusting performance. Amasyali et al. [11], for example, reported shorter procedural and lasing times using 150 μm fibers compared to 200 μm fibers in a 3D kidney model.

Our findings partially align with those of Panthier et al. [10], who observed higher ablation rates with larger TFL fibers. Similarly, we found that the 200 μm TFL fiber achieved greater AV than the 150 μm fiber, although this reached statistical significance only at 1 J × 20 Hz. However, unlike Panthier et al., we did not observe superior ablation rates of TFL-150 μm compared to Ho: YAG systems, possibly due to the use of newer pulse-modulated high-power Ho: YAG platforms in our study.

Interestingly, the performance of TFL-150 μm appeared to depend on pulse settings and was characterized by broader and shallower ablation profiles, particularly at 1 J × 20 Hz, where wider fissures were achieved despite similar or lower DOF compared to most Ho: YAG systems. Although some lasers achieved higher AV, this was frequently associated with deeper penetration. These findings suggest that TFL-150 μm may produce an ablation geometry potentially favorable for dusting by promoting progressive surface erosion and fine particle generation while limiting deep focal excavation.

Additionally, with Magneto, lower energy settings (1 J and 0.3 J compared to 1.5 J) tended to produce greater WOF rather than greater DOF. Despite TFL consistently producing higher WOF compared to the other lasers, DOF was comparable in some cases and even lower in others, particularly in the comparisons against Magneto. This may be explained by the lower peak power generated at lower energies with Magneto, potentially making this technology suitable for dusting techniques. On the contrary, Cyber Ho without Magneto tended to produce higher DOF and lower WOF at 1 J × 20 Hz and 0.3 J × 50 Hz, potentially favoring fragmentation behavior, even when using classically described “dusting” settings. It is worth noting a recent in vivo prospective controlled trial comparing TFL and a pulse-modulated high-power Ho: YAG laser, which reported no significant differences in stone-free rates or laser efficiency [35]. Whether our findings can be translated into clinical practice remains an open question and warrants further investigation.

A potential explanation for our findings lies in the intrinsic physical differences between TFL and Ho: YAG lasers. TFL operates at a wavelength (1,940 nm) closer to the peak absorption of water than Ho: YAG (2,120 nm), resulting in substantially greater water absorption and potentially more efficient energy delivery to the stone [36].

Although we standardized the fiber-to-stone distance at 0.5 mm throughout all experiments, crater morphology evolves dynamically during ablation and may progressively increase the effective working distance as fissures deepen. Under these conditions, Ho: YAG lasers tended to generate deeper and narrower ablation profiles, whereas TFL produced broader and more superficial patterns. These distinct ablation geometries may influence energy transmission within the crater, particularly as increasing depth and beam divergence may reduce effective energy delivery in Ho: YAG systems. Conversely, the more homogeneous energy distribution of TFL may allow more consistent superficial ablation despite changes in crater geometry.

Therefore, even when using pulse-modulated Ho: YAG systems with extended pulse durations and reduced peak power, inherent wavelength-related differences may limit their ability to fully reproduce the ablation characteristics observed with TFL [24].

From a clinical perspective, these physical differences translate into distinct surgical behaviors: Ho: YAG remains highly effective for fragmentation due to its high peak power, while TFL may offer advantages for dusting-related strategies due to its broader and shallower ablation geometry, reduced retropulsion, improved visibility, and compatibility with smaller fibers, which may facilitate miniaturization strategies in f-URS.

### Limitations and future directions

This study is not without limitations. The use of BegoStone phantoms instead of human stones may have influenced our results. Some authors argue that these artificial stones do not accurately replicate natural kidney stones [19], while others suggest that they can reasonably match the physical properties of various stone compositions [37]. Human stones present significant heterogeneity in composition, internal structure, porosity, and water content, which may influence laser–stone interaction, energy absorption, and crack propagation patterns. These differences may affect ablation behavior and fragmentation efficiency, limiting the direct translation of in vitro findings to clinical practice. Nevertheless, BegoStones offer a reasonable alternative by providing homogeneity across experiments.

Other limitations include the lack of measurement of stone movement and retropulsion. In clinical scenarios, retropulsion may significantly affect energy delivery and ablation efficiency, particularly with high peak power Ho: YAG pulses. Moreover, fiber degradation, burnback effects, carbonization, and debris accumulation were not evaluated. In clinical practice, progressive fiber tip degradation may alter energy transmission, reduce efficiency, and modify ablation patterns over time, particularly in higher-peak-power systems during prolonged laser use. To diminish these effects, the fiber tip was cut before every experiment. These factors should be considered when translating in vitro findings to clinical practice.

Despite these limitations, to the best of our knowledge, this is the first study to compare TFL with the latest advancements in Ho: YAG laser technology, while also evaluating their performance across different fiber sizes. Further in vivo and clinical studies are required to validate these findings.

## Conclusions

In this in vitro study, TFL with 200 μm fiber achieved higher AV than Ho: YAG in most settings, although statistically significant differences were observed in only one-third of comparisons. The TFL-150 μm demonstrated similar AV to Ho: YAG in most settings, with the few observed differences primarily related to greater DOF achieved by some Ho: YAG modalities rather than differences in WOF. Beyond AV, the TFL-150 μm may offer additional advantages in terms of irrigation flow, visibility, ureteroscope tip deflection, and compatibility with miniaturized f-URS and aspiration systems, supporting its potential applicability for dusting strategies while maintaining comparable AV.

## Data Availability

No datasets were generated or analysed during the current study.
